# Enhancing Query Formulation for Universal Image Segmentation

**DOI:** 10.3390/s24061879

**Published:** 2024-03-14

**Authors:** Yipeng Qu, Joohee Kim

**Affiliations:** Department of Electrical and Computer Engineering, Illinois Institute of Technology, Chicago, IL 60616, USA; yqu13@hawk.iit.edu

**Keywords:** computer vision, image segmentation, semantic segmentation, panoptic segmentation, transformer

## Abstract

Recent advancements in image segmentation have been notably driven by Vision Transformers. These transformer-based models offer one versatile network structure capable of handling a variety of segmentation tasks. Despite their effectiveness, the pursuit of enhanced capabilities often leads to more intricate architectures and greater computational demands. OneFormer has responded to these challenges by introducing a query-text contrastive learning strategy active during training only. However, this approach has not completely addressed the inefficiency issues in text generation and the contrastive loss computation. To solve these problems, we introduce Efficient Query Optimizer (EQO), an approach that efficiently utilizes multi-modal data to refine query optimization in image segmentation. Our strategy significantly reduces the complexity of parameters and computations by distilling inter-class and inter-task information from an image into a single template sentence. Furthermore, we propose a novel attention-based contrastive loss. It is designed to facilitate a one-to-many matching mechanism in the loss computation, which helps object queries learn more robust representations. Beyond merely reducing complexity, our model demonstrates superior performance compared to OneFormer across all three segmentation tasks using the Swin-T backbone. Our evaluations on the ADE20K dataset reveal that our model outperforms OneFormer in multiple metrics: by 0.2% in mean Intersection over Union (mIoU), 0.6% in Average Precision (AP), and 0.8% in Panoptic Quality (PQ). These results highlight the efficacy of our model in advancing the field of image segmentation.

## 1. Introduction

As a key branch of computer vision, image segmentation aims to partition an image into distinct segments, each representing a specific category. There are three tasks in image segmentation. Semantic segmentation classifies each pixel by category, and instance segmentation identifies and segments objects within each instance mask. Panoptic segmentation combines both semantic and instance segmentation to provide a more comprehensive understanding of the visual scene.

One of the popular approaches for semantic segmentation is an encoder–decoder architecture. The encoder is to extract multi-scale features from images, and the decoder produces per-pixel-level predictions. Encoders based on the Convolutional Neural Network (CNN) [1,2,3,4] have been dominant in semantic segmentation with cascading convolutional layers. They use small convolutional kernels to balance computational efficiency with the need for a large receptive field. However, there is a limit to the number of layers, and over-stacking can lead to training saturation. To address this, transformers [5] for Natural Language Processing (NLP) tasks have been introduced to computer vision. To process images, vision transformers divide them into fixed-size patches and flatten them into 1D arrays. Most importantly, they can offer superior representation learning than CNNs by extracting long-range dependencies in parallel.

In simpler terms, it was previously believed that mask classification was only apt for instance segmentation, while semantic segmentation required per-pixel-classification. However, MaskFormer [6] demonstrated that mask classification can effectively handle both segmentation types with the same model structure and training process. As its successor, Mask2Former [7] enhanced segmentation accuracy by incorporating mask attention to the transformer decoder. However, the improvement leads to the expense of higher computational costs and latency. While purporting to offer universal image segmentation, MaskFormer [6] and Mask2Former [7] necessitate separate training for distinct tasks. In contrast, OneFormer [8] stands out as a genuinely universal framework. It achieves state-of-the-art performance across three image segmentation tasks through a single training cycle. This is made feasible by employing a joint training strategy coupled with a task-conditioned query formulation. These queries begin as task token repetitions and are subsequently refined by a two-layer transformer. With the involvement of the image features, the transformer decoder produces task-dependent and image-aware outputs. In the quest to refine segmentation accuracy, a specialized module is introduced during training for the optimization of queries. This module generates a group of text queries (denoted as $Q_{text}$) to accentuate inter-class differences by guiding the object queries using a query-text contrastive loss. The text is derived from the training dataset, where each sentence corresponds to one object class present in an image. Due to the mismatch between the average count of objects per image and the number of object queries, the text lists are padded with a substantial number of duplicated sentences that carry limited supervisory information.

However, this universal segmentation method faces two primary issues that impede efficient query optimization during training. First, the generation of the text list entails a significant amount of redundant information. With the default number of object queries set to either 150 or 250 based on the choice of backbone, the majority of sentences contribute minimal supervisory information. This leads to excess parameters in the generation of $Q_{text}$ and increased computational costs during training. Second, the fixed one-to-one matching between $Q_{text}$ and *Q* is not the most effective method for computing contrastive loss because it does not fully account for the multifaceted roles of *Q* in image segmentation. The ablation studies of MaskFormer [6] suggest that object queries, interpreted as region proposals, can capture objects from different categories. Moreover, the distribution of unique classes each query can recognize is not uniform. In contrast, each text query in $Q_{text}$ is linked to a specific class or object within an image, which differs fundamentally from object queries. Therefore, the one-to-one matching mechanism in contrastive loss computation restricts object queries’ capability to learn more robust representations. The experimental results in Section 4.4.1 show that our attention-based contrastive loss boosts the model’s performance, independent of text formulation methods.

To overcome these limitations, we present an Efficient Query Optimizer (EQO) incorporating efficient text generation and attention-based contrastive loss. We streamline the text list by consolidating all semantic prompts into a single sentence per image. This strategy preserves essential inter-class and inter-task information only. Additionally, we employ an attention mechanism to establish a one-to-many matching relationship for contrastive loss computation. In this way, more adaptive matching is feasible in the query optimization stage. In addition, the proposed method can be extended to scenarios involving multiple sensors such as multimodal scene understanding in autonomous driving. In order to achieve robust and accurate scene understanding in autonomous driving, autonomous vehicles are usually equipped with multimodal sensors, (e.g., cameras, LiDARs, and Radars), and different sensing modalities can be fused to exploit their complementary properties. For example, disparity maps, as derived from the depth maps captured by stereo cameras [9], can be used as input to a Transformer to generate depth embeddings and used to learn a joint embedding space between images and disparity maps using similar approaches proposed in [10]. Specifically, the image embeddings generated by our proposed model can be paired with the depth embeddings generated by a plug-in module for contrastive learning during training. Based on this approach, our proposed model can be extended into applications using multiple sensors without changing the base architecture.

Our major contributions are summarized as follows:

- In this study, we introduce an Efficient Query Optimizer (EQO) designed for universal image segmentation. This optimizer is adept at directing object queries towards capturing task-dependent and object-centric information, which is extracted from input images.
- Our research addresses the issue of redundant text formulation observed in the existing methods. The text paradigm we propose includes only the information of the objects present in the images. To maintain the capability of a single object query to recognize objects of multiple classes, we depart from the traditional contrastive loss with its fixed one-to-one matching mechanism. Instead, we implement an attention-based loss computation strategy, which inherently supports a one-to-many matching process during training.
- We evaluate our model across three segmentation tasks (semantic-, instance-, and panoptic segmentation) on two datasets (ADE20K [11] and Cityscapes [12]) with the Swin-T Backbone. Our model outperforms its baseline [8] and other Swin-T-based models on three image segmentation tasks; on the ADE20K dataset [11], our model achieves **49.2 mIoU** (single-scale), **29.3 AP**, and **43.6 PQ** using one universal architecture. On the Cityscapes dataset [12], the presented architecture achieves **81 mIoU** (single-scale), **41.9 AP**, and **65.6 PQ**.

## 2. Related Work

### 2.1. Transformer-Based Image Segmentation

Transformers [5] were initially designed for Natural Language Processing. They have been effectively adapted for computer vision to learn global patterns in visual data. ViT [13] divides images into patches, in order to process them as 1D sequences. Enhancements like the ViT Adaptor [14] introduce modules for local feature extraction without changing ViT’s structure. It improves feature refinement through cross-attention in Multi-Scale Feature Extractors. However, vision transformers often require substantial data. Addressing this, the Data-efficient image Transformer (DeiT) [15] employs knowledge distillation to mimic larger models with less data. The Swin Transformer [16,17] introduces patch merging for multi-scale features and a window-based self-attention for high-resolution images. For real-time needs, the Lightweight Vision Transformer (LeViT) [18] provides a faster alternative with simplified self-attention and distillation. This achieves a balance between efficiency and performance. These developments highlight the versatility of transformers in bridging language and image processing.

**Semantic Segmentation.** Semantic segmentation is a significant task in the domain of computer vision. It involves the partitioning of images into distinct regions, with the goal of labeling each pixel with a class from a predefined set of categories. SETR [19] is a ViT-based model for semantic segmentation. It employs a decoder that aggregates multi-level features via pixel-wise addition. However, its efficiency in capturing different-scale objects is limited due to shared resolution. SegFormer [20] improves upon this by modifying the ViT-based encoder to obtain multi-scale features. SeMask [21] suggests that they do not fully utilize image context, so it introduces SeMask Attention blocks. These blocks can be easily integrated into a transformer-based backbone, which enables more effective context incorporation.

**Instance Segmentation.** Instance segmentation is a sophisticated technique in the field of computer vision that involves two key steps: object detection and mask-classification. Unlike semantic segmentation, which labels each pixel of an image without differentiating between individual objects of the same class, instance segmentation goes a step further by identifying each specific instance of an object within the same class and assigning class labels to each of them. As an end-to-end transformer-based model for object detection, DETR [22] achieves instance segmentation by adding a mask head. To address DETR’s issue with slow convergence and further improve its performance, the following research [23,24] presents different solutions.

**Panoptic Segmentation.** Panoptic segmentation [25] is a technique in computer vision that merges the principles of semantic segmentation and instance segmentation. By doing so, it offers a comprehensive perspective of an image. It recognizes all distinct objects (“things”) and background elements (“stuff”) in the scene. Various models have been developed to address this task effectively. Models such as Panoptic FPN [26] and Panoptic-DeepLab [27] utilize a shared backbone network for both semantic segmentation and object detection. However, they differentiate between “things” and “stuff” by employing two separate segmentation heads. The Unified Panoptic Segmentation Network (UPSNet) [28] features a unique Panoptic Head module that integrates the outputs from the instance and semantic segmentation heads. It introduces an innovative Instance-Upsampling module to maintain consistency between instance and semantic segmentation. EfficientPS [29] merges EfficientNet [30] and EfficientDet [31] into a single network. This combination makes it a potent yet resource-efficient tool for panoptic segmentation.

### 2.2. Universal Segmentation

Universal segmentation aims to create a single but versatile model capable of performing various types of image segmentation tasks. Compared to traditional segmentation models, a universal segmentation model is designed to handle all these tasks simultaneously, streamlining the process and reducing the need for multiple specialized models. MaskFormer [6] and Mask2Former [7] excel in universal segmentation because they adopt a mask-classification approach and can handle a variety of segmentation tasks seamlessly. MaskFormer [6] consists of three primary components: a backbone for feature extraction from input images, a pixel-decoder for refining and upsampling these features, and a transformer decoder module. This decoder generates predicted labels and a set of per-segment embeddings, with the number of embeddings being equal to the number of object queries. Building upon MaskFormer [6], Mask2Former [7] introduces an innovative approach by incorporating mask attention into the Transformer Decoder module. This enables Mask2Former [7] to achieve more significant advancements in universal image segmentation than its predecessor [6].

However, both these models require separate training phases for different segmentation tasks. Addressing this limitation, OneFormer [8] introduces a more streamlined approach. It achieves universal segmentation with a single training process by using a task token to condition the object queries. Furthermore, OneFormer [8] employs a query-text contrastive loss to effectively learn the differences between tasks and categories. As a result of these innovations, OneFormer [8] demonstrates superior performance in universal image segmentation with just one training cycle. This advancement signifies a significant step forward in the efficiency and versatility of image segmentation models.

## 3. Proposed Method

### 3.1. Preliminary

OneFormer [8] proposes a universal image segmentation framework based on vision transformers that can be trained only once with a single model. This method assigns a random task (either semantic, instance, or panoptic) to each image in a training batch. The model recognizes the specific task through a designated template sentence, “*The task is {TASK}*”. {TASK} is uniformly sampled from the three segmentation types. During training, each image is prepared with the appropriate ground truth corresponding to its assigned task. The training procedure starts with the tokenization of the task input, and then the tokenized sequence is input to a two-layer multilayer perceptron (MLP) to generate $Q_{task}$. $Q_{task}$ conditions the object queries by initializing them as repetitions of $Q_{task}$. Through a two-layer transformer decoder, these object queries interact with a feature map produced by the pixel decoder. The final step involves combining $Q_{task}$ with the N−1 queries to create a total of *N* object queries.

**Figure 1:**
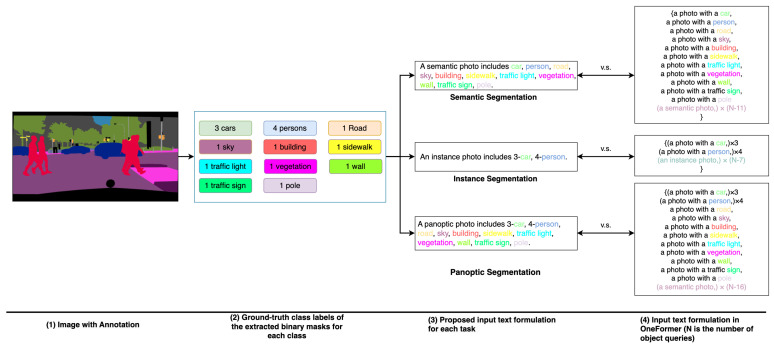
Our Efficient Text Formulation. The proposed method creates different template sentences for each of the three segmentation tasks. Compared to our efficient design, the text produced by OneFormer exhibits greater redundancy.

In order to enhance the differentiation among different segmentation tasks and classes, a query-text contrastive loss is incorporated during training, which employs a one-to-one matching strategy for similarity learning. It compares *N* object queries (denoted as *Q*) with *N* text-based queries (named $Q_{text}$). To generate $Q_{text}$, a set of binary masks for each category presented in an input image is extracted based on the task-specific GT labels. Then, as shown in the last column of Figure 1, a list of text *T* with a template “*a photo with a* {CLS}” is created, where {CLS} is the class name of the binary mask. Since the number of binary masks per image varies over the dataset and is much less than the predefined number of object queries *N*, *T* is padded with “*a/an {TASK} photo*” to generate a padded list $T_{pad}$ with a constant length *N*. $T_{pad}$ is processed with a text encoder [32] to generate $Q_{text}$, which is used to compute a query-text contrastive loss.

### 3.2. Architecture Overview

**Figure 2:**
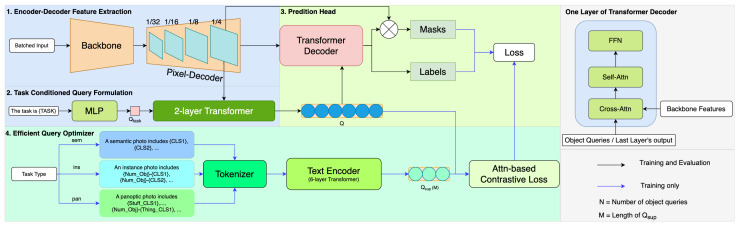
Model Architecture. We present an innovative Efficient Query Optimizer that uses text data to refine the segmentation process. This optimizer is inactive during the inference to boost efficiency. Our model’s unique design allows for precise image segmentation with low computational needs and minimal parameters, requiring only a single training session.

The network consists of four components: the encoder–decoder feature extractor with a Swin Transformer backbone and a pixel decoder; the task-conditioned query formulation module; the prediction head with a Transformer decoder; and our presented Efficient Query Optimizer (EQO). Figure 2 shows the overall architecture of the proposed model. The Swin Transformer-based backbone extracts multi-scale feature maps from an input image, and then the pixel decoder progressively upsamples the feature maps to generate more detailed and higher-resolution feature maps. To realize the goal of universal image segmentation with a single training, we employ the query formulation method used in OneFormer [8], which generates $Q_{task}$ from a pre-defined task input. The object queries are initialized as the repetitions of $Q_{task}$. Inside a two-layer transformer decoder, the interaction between object queries and the pixel decoder’s feature map is measured in the cross-attention calculation. This collaborative mechanism facilitates the generation of task-sensitive queries essential for universal image segmentation.

Different from existing methods, we propose an Efficient Query Optimizer (EQO) to enhance the query formulation process. The main function of EQO is to create supervisory queries called $Q_{sup}$ with task- and class-dependent information in an efficient manner. EQO first extracts task-sensitive semantic information from an image and produces a single template sentence, as shown in Figure 1. After tokenization, a text encoder [32] is applied to encode the text tokens and generate the supervisory queries $Q_{sup}$. Since the sequence length *M* of $Q_{sup}$ is much smaller than the number of object queries *N*, the computational complexity is reduced significantly. However, the conventional query-text contrastive loss cannot be used in the asymmetric scenario. Therefore, we propose an attention-based contrastive loss to measure the similarity between the object queries *Q* and the supervisory queries $Q_{sup}$ during training, which avoids the one-to-one matching approach in the regular contrastive loss. Since the proposed EQO is used only for training and can be discarded during inference, our proposed method can improve the efficiency of query formulation without additional computation costs at inference. Finally, a transformer decoder is used to obtain the task-dynamic class and mask predictions.

### 3.3. Enhancing Query Representations

#### 3.3.1. Improving Input Text Formulation

In OneFormer [8], the text lists formulated for the universal image segmentation task are not efficient. The main issue is that a large portion of the text data does not contribute to identifying objects within an image. The inefficient formulation of text lists increases computational costs and requires unnecessary parameters. Our goal is to summarize the fundamental semantic content of an image in a brief template sentence that includes the information of all the binary masks extracted from the GT label without losing the ability to identify different classes or tasks. As shown in Figure 1, the proposed method creates a task-specific template sentence from an image by considering the features of each segmentation task. Specifically, to address the semantic segmentation task, we form a template sentence including “things” and “stuff” classes without specifying the quantities of “things” classes. For instance segmentation, the proposed template sentence includes the list of “things” classes with the corresponding quantities. Since panoptic segmentation combines the features of semantic and instance segmentation, the proposed template sentence for panoptic segmentation includes a list of “stuff” classes with class labels only and “things” classes with class labels and quantities.

In situations where the ground truth of an image reveals that there are no foreground objects (which belong to categories of interest), the text input is formulated as “*A {TASK} photo includes nothing*”(named no-object indicator). This template is used even though such images are excluded during the inference process. This specific text template helps the model accurately identify and minimize incorrect labeling. By explicitly stating the absence of foreground objects in the training phase, the model learns to better distinguish between the presence and absence of relevant objects, thereby enhancing its overall accuracy. The efficacy of the no-object indicator is proven in Section 4.4.3, where it improves AP by 0.3% and mIoU by 0.1%.

#### 3.3.2. Attention-Based Contrastive Loss

It is found that object queries are able to recognize multiple categories with distinct semantic meanings in image segmentation. However, in OneFormer [8], one text query of $Q_{text}$ corresponds to a single object category presented in an image, and it is matched to one object query during the training stage. This one-to-one contrastive loss between object queries *Q* to $Q_{text}$ impedes *Q*’s ability to learn robust representations. On the other hand, our proposed input text formulation results in a discrepancy between the number of supervisory queries *M* and the count of object queries *N*. It is not practical to use conventional contrastive loss in this situation.

**Figure 3:**
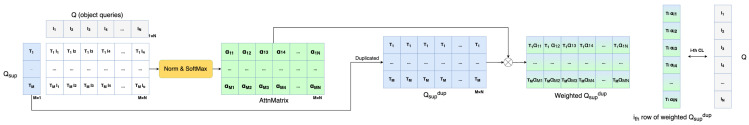
Attention-based contrastive loss. One-to-many contrastive loss is computed between object queries *Q* and each text query (denoted as $T_{i}$) in supervisory queries $Q_{sup}$.

To address these issues, an attention-based one-to-many matching approach for contrastive loss is presented between object queries *Q* and $Q_{sup}$, as shown in Figure 3. The text encoder processes the proposed input text template and then generates $Q_{sup}$. The similarity between *Q* and $Q_{sup}$ is measured by performing multiplication. Following normalization and SoftMax to the previous result, an attention matrix (denoted as AttnMatrix) is formed with each element representing a normalized attention score. Then each text query $T_{i}$ in $Q_{sup}$ is duplicated by *N* times. The contrastive loss (CL) is computed between object queries *Q* and *N* copies of $T_{i}$, with the attention scores serving as weights. The remaining work is to repeat the previous steps for *M* times, each time using a different text query. Notably, this process does not significantly increase computational demands since *M* is substantially small compared to *N*.

The overall procedure is shown in Algorithm 1. For simplicity, the batch size and the channel number are omitted in Figure 3 and Algorithm 1.
**Algorithm 1** Pseudocode of Attention-Based Contrastive Loss Computation. **Input 1:** $Q_{sup}\in R^{M\times 1}$                   ▹ M: number of $Q_{sup}$ **Input 2:** $Q\in R^{N\times 1}$                       ▹ N: count of *Q* **Output:** $loss^{cl}$ $AttnMatrix\in R^{M\times N}\leftarrow SoftMax(Norm\left(Q_{sup}⊙Q^{T}\right)$   ▹⊙: Matrix Multiplication $Q_{sup}^{dup}\in R^{M\times N}\leftarrow Duplicate\left\{Q_{sup}\right\}$ $loss^{cl}\leftarrow 0$ **for** i∈{0,1,2,...,M−1}
**do**     $T_{i}\in R^{N\times 1}$                         ▹ i-th row of $Q_{sup}^{dup}$     $attn_{i}\in R^{N\times 1}$                     ▹ i-th row of AttnMatrix     $loss_{i}^{cl}\leftarrow CL_{(attn_{i}\times T_{i})↔Q}$    ▹ CL: Contrastive Loss; ×: element-wise multiplication     $loss^{cl}\leftarrow loss^{cl}+loss_{i}^{cl}$ **end for** **return** $loss^{cl}\leftarrow \frac{1}{M}\cdot loss^{cl}$


In the loop of Algorithm 1, the contrastive loss is computed between each $T_{i}$ in $Q_{sup}^{dup}$ to object queries *Q* by “CL”. Equations (1) to (3) demonstrate the details of this procedure. Considering that the batch size is *B*, there are *B* pairs of object queries and supervisory queries in total. Specifically, $Q_{k}$ denotes *N* object queries of the $k_{th}$ pair, and ${\left(attn_{i}\times T_{i}\right)}_{k}$ refers to the $i_{th}$ weighted text query of the $k_{th}$ group of supervisory queries.

(1) $$\begin{array}{cc} CL_{Q\to (attn_{i}\times T_{i})} & =-log\frac{exp(Q^{T}⊙\left(attn_{i}\times T_{i}\right))}{\sum_{k=1}^{B}exp\left(Q^{T}⊙{\left(attn_{i}\times T_{i}\right)}_{k}\right)}, \end{array}$$

(2) $$\begin{array}{cc} CL_{(attn_{i}\times T_{i})\to Q} & =-log\frac{exp({\left(attn_{i}\times T_{i}\right)}^{T}⊙Q)}{\sum_{k=1}^{B}exp\left({\left(attn_{i}\times T_{i}\right)}^{T}⊙Q_{k}\right)}, \end{array}$$

(3) $$\begin{array}{cc} CL_{(attn_{i}\times T_{i})↔Q} & =CL_{Q\to (T_{i}\times attn_{i})}+CL_{(T_{i}\times attn_{i})\to Q} \end{array}$$

## 4. Experimental Results

### 4.1. Datasets

We measure our model’s performance on two widely-used datasets: ADE20K [11] and Cityscapes [12]. The ADE20K dataset [11] is a richly annotated dataset extensively used in computer vision research. It contains over 20,000 images spanning diverse scenes, with pixel-level annotations for more than 150 object categories, making it ideal for tasks such as semantic and instance segmentation. The Cityscapes dataset [12] is designed for image segmentation tasks, specifically focused on urban street driving scenarios. It comprises 5000 images captured across 50 cities and labeled with 19 semantic classes. The dataset is split into a training set with 2975 images, a validation set with 500 images, and a test set comprising 1525 images.

We utilize three key metrics for assessing our model: mean Intersection-over-Union (mIoU) [33] for semantic segmentation, Panoptic Quality (PQ) [25] for panoptic segmentation, and Average Precision (AP) [34] for instance segmentation. PQ is a comprehensive measure that evaluates a model’s performance on both background elements (stuff) and distinct objects (things) simultaneously. PQ=SQ×RQ. Segmentation Quality (SQ) measures the mIoU over true-positive predictions, assessing how accurately the model identifies and segments objects. Recognition Quality (RQ), on the other hand, is the harmonic mean of Precision and Recall, which indicates how effectively the model produces correct predictions.

### 4.2. Implementation Details

In our model, we employ the Swin Transformer [16] as the backbone, and we test our model based on one Swin variant: Swin-T. The Backbone is pretrained on the ImageNet-1k dataset with an image resolution of 224 × 224. For the ADE20K [11] and Cityscapes [12] datasets, the input images are cropped to sizes of 512 × 512 and 512 × 1024, respectively. Given that OneFormer [8] does not report performance metrics using the Swin-T backbone, we have undertaken the task of reproducing these results with two NVIDIA GeForce RTX 3090. Our reproduction uses a batch size of 12 for the ADE20K dataset and 8 for the Cityscapes dataset. To ensure a fair comparison, we train our model with the same batch size. Our model is built with the PyTorch (1.10.1) [35] framework and the Detectron2 (v0.6) [36] library. We utilize the AdamW [37] optimization algorithm, setting the base learning rate to 0.00009 for the ADE20K dataset and to 0.00007 for the cityscapes dataset. In our model, BertTokenizer [38] is employed to tokenize all the text input. In addition, we unify the tokenizer when reproducing the performance results of the baseline [8] in a fair way.

### 4.3. Experimental Results

#### 4.3.1. ADE20K

Table 1 showcases an evaluation of our model on the ADE20K [11] dataset, which benchmarks its performance against other competitive models in the domain of universal segmentation with comparable parameter volumes. Each model listed in Table 1, including ours, is trained using images with a resolution of 512×512. Notably, during inference, our model’s EQO component is removed, resulting in a network structure identical to that of the baseline model [8]. Therefore, a direct comparison of the net parameter count is performed between our model and OneFormer [8] during the training phase. Furthermore, the computation of GFLOPs is measured during the evaluation stage.

Our methodology stands out for its significant reduction in parameter complexity, decreasing by 4.9 million parameters compared to our baseline model [8]. Additionally, our model demonstrates a higher throughput, which processes 0.5 more images per second than OneFormer [8] during training. More importantly, these enhancements in efficiency do not compromise performance; on the contrary, our model shows improved performance across all three segmentation tasks when compared to the baseline models [8]. Specifically, our model outperforms OneFormer [8] by 0.2% in mean Intersection over Union (mIoU), 0.8% in Panoptic Quality (PQ), and 0.6% in Average Precision (AP). When compared with other models [6,7] in the universal segmentation domain, our model’s performance advantage is even more pronounced. The visualization results can be found in Appendix B.

#### 4.3.2. Cityscapes

In Table 2, we validate our model’s performance across three tasks on the Cityscapes dataset and compare it to other competitive models in universal image segmentation. The crop size of training images is 512×1024 for the models shown in Table 2 (except SeMask [21] using 768×768 images). Notably, with a more efficient network architecture, our model is superior to its baseline [8] on semantic and panoptic segmentation by 0.3% and 0.7%, respectively.

### 4.4. Ablation Study

The analysis of our model is performed using Swin-T backbone on the ADE20K [11] dataset.

#### 4.4.1. Attention-Based Contrastive Loss

To evaluate the effectiveness of our attention-based contrastive loss, we only replaced the contrastive loss in OneFormer [8] with ours, as detailed in Table 3’s second row. This substitution led to a 0.2% improvement in Panoptic Quality (PQ) and a 0.4% increase in mean Intersection over Union (mIoU) while maintaining similar Average Precision (AP) in instance segmentation. This proves that a more flexible contrastive loss calculation can improve performance, and this enhancement is independent of the text input format used in the model.

#### 4.4.2. Efficient Text Paradigm

In the third row of Table 3, we show the performance boost from our text paradigm, which enhances parameter efficiency and reduces complexity. Paired with the attention-based contrastive loss, this approach leads to a significant accuracy improvement, with a 0.6% increase in Panoptic Quality (PQ) and a 0.7% rise in Average Precision (AP), compared to using only our attention-based contrastive loss. By reformatting text input to minimize redundancy, the Efficient Query Optimizer is proven to provide a more effective and targeted supervision for object queries, resulting in notable performance gains.

#### 4.4.3. No-Object Indicator

In Section 3.3.1, we introduce the “no-object indicator” to address images lacking relevant objects, using the template “*A/An {TASK} photo includes nothing*”. This approach is aimed at reducing misclassification by enhancing the model’s ability to discern the absence of target objects within images. Table 4 presents a comparative analysis of the performance metrics across different models: the baseline [8], our proposed model, and a variant of our model excluding the no-object indicator. The results demonstrate our model’s improvement over the model variant without the no-object indicator, with a 0.3% increase in Average Precision (AP) and a 0.1% enhancement in mean Intersection over Union (mIoU).

#### 4.4.4. Number of Text Queries in $Q_{sup}$

Table 5 showcases the performance comparison among variants of our model, each with a different number of text queries *M*. In our model, the number of text queries aligns with the number of tokens in a template sentence. To investigate the impact of different *M* values, we set *M* to 1. In this scenario, a single text query corresponds to a template sentence containing semantic information about all objects in an image. While this approach minimizes computational costs, it results in a performance decline across all segmentation tasks: a decrease of 0.8% in mean Intersection over Union (mIoU), 0.4% in Average Precision (AP), and 0.7% in Panoptic Quality (PQ). However, our model still outperforms the baseline [8] in PQ by 0.1% and in AP by 0.2%, with M equal to 1.

#### 4.4.5. Modality Fusion in EQO

Object queries are visual-based representations, which are regarded as region proposals. On the other hand, $Q_{sup}$ is generated solely by the text-based input. We try to investigate how the differences between modalities affect prediction accuracy. To facilitate this, we introduce visual features from the pixel-decoder into EQO using a cross-modal skip-connection network from mPLUG [41]. This network comprises a TextDecoder (a single-layer Transformer Decoder) and a FusionEncoder (a single-layer Transformer Encoder).

While mPLUG [41] utilizes single-scale visual features, our pixel-decoder produces visual features at multiple levels. To assess the effect of these multi-scale features on cross-modality fusion, we set up two distinct experiments: one utilizing single-scale features and the other employing multi-scale features. Further details on these setups and the architecture of the cross-modal skip-connection can be found in Appendix A.

According to Table 6, our model performs best without fusion operations, particularly in PQ and AP metrics. We suspect the performance drop with fusion operations is due to the non-pre-trained parameters of the cross-modal skip-connection network, which makes the training more complex and potentially decreases accuracy. Further investigation into this aspect is planned in future research.

## 5. Limitations

Cross-modality models [42,43] have proved that learning general representation from different modalities can also benefit single-modality downstream tasks. Our model processes text data through a distinct branch, separate from the visual input processing modules. Through an ablation study, we explored the effect of combining textual and visual modalities within the Efficient Query Optimizer (EQO). Contrary to popular belief, our findings reveal that this modality fusion does not lead to better segmentation accuracy. We hypothesize that this unexpected outcome may be attributed to the lack of pre-training for the cross-modality fusion module, which potentially complicates the training process and adversely affects accuracy. Future experiments are planned to further investigate the potential benefits of cross-modality understanding in image segmentation tasks.

Furthermore, our model surpasses the benchmark model [8] in Average Precision (AP) by 0.6% on the ADE20K dataset. However, the performance gap between our model and the OneFormer [8] on the Cityscapes dataset is minimal when evaluated using the same metric. We speculate that this minimal difference is attributed to the higher average number of instances in the Cityscapes dataset compared to ADE20K, suggesting that the advantages of our model in instance segmentation exhibit diminishing returns in challenging scenarios.

## 6. Conclusions

In this paper, we introduce the Efficient Query Optimizer (EQO), an efficient approach to universal image segmentation. It efficiently employs multi-modal data to refine query optimization during training. Inside this module, attention-based contrastive loss is presented with one-to-many matching, which enhances the capability of object queries to capture multiple categories within images. To avoid redundancy in the text input, we redesign the text template for extracting semantic information from input images, which achieves a dual benefit of computational efficiency and improved performance. Notably, the components responsible for query optimization are not required during the inference stage, allowing for a more parameter-efficient learning process. Comprehensive experiments have been conducted to validate our model’s superior performance across all three segmentation tasks, compared to OneFormer and other universal segmentation models. We hope our work will stimulate further research interest in the area of query optimization for universal image segmentation, paving the way for advancements in this field.

## Figures and Tables

**Table 1:** Image segmentation on ADE20K val with 150 categories. The single-scale mIoU is reported.

Method	Backbone	mIoU (s.s.)	PQ	AP	#Params	GFLOPs	Throughput
***Individual Training***							
Swin-UperNet [16,39]	Swin-T ^†^	46.1 *	-	-	60.0 M	236.0	-
Segmenter [40]	DeiT-B	48.7	-	-	86.0 M	-	-
MaskFormer [6]	Swin-T ^†^	46.7	-	-	42.0 M	55.0	-
	R101	45.5	-	-	60.0 M	73.0	-
Mask2Former [7]	Swin-T ^†^	47.7	-	-	47.4 M	74.0	-
	R101	47.8	-	-	63.0 M	90.0	-
SeMask [21]	Swin-S ^‡^	45.9	-	-	56.0 M	63.0	-
***Joint Training***							
OneFormer [8]	Swin-T ^†^	49.0	42.8	28.7	68.3 M	81.4	16.0 img/s
Our Model	Swin-T ^†^	**49.2**	**43.6**	**29.3**	63.4 M	81.4	16.5 img/s

†: Backbone is pretrained on ImageNet-1k; ‡: backbone is pretrained on ImageNet-22k with 384×384 images; and *: multi-scale mIoU. Numbers in bold represent the best performance in each metric.

**Table 2:** Image Segmentation on Cityscapes val. The single-scale mIoU is reported.

Method	Backbone	mIoU (s.s.)	PQ	AP	#Params	GFLOPs	Throughput
***Individual Training***							
Segmenter [40]	DeiT-B	80.6	-	-	86.0 M	-	-
SETR-PUP [19]	ViT-L	79.3	-	-	318.3 M	-	-
Mask2Former [7]	Swin-T ^†^	**82.1**	63.9	39.7	47.4 M	-	-
	R101	80.1	62.4	38.5	63.0 M	-	-
SeMask [21]	Swin-S ^‡^	77.1	-	-	56.0 M	134.0	-
***Joint Training***							
OneFormer [8]	Swin-T ^†^	80.7	64.9	41.9	68.3 M	168.2	6.6 img/s
Our Model	Swin-T ^†^	81.0	**65.6**	**41.9**	63.4 M	168.2	7.9 img/s

†: Backbone is pretrained on ImageNet-1k; ‡: backbone is pretrained on ImageNet-22k with 384×384 images. Numbers in bold represent the best performance in each metric.

**Table 3:** Ablation on Attention-based contrastive loss and efficient text paradigm. Numbers in bold represent the best performance in each metric.

	PQ	mIoU	AP
Baseline [8]	42.8	49.0	28.7
+Our Contrastive Loss	43.0	**49.4**	28.6
+Our Efficient Text Paradigm	**43.6**	49.2	**29.3**

**Table 4:** Ablation on no-object indicator. Numbers in bold represent the best performance in each metric.

	PQ	mIoU	AP
Baseline [8]	42.8	49.0	28.7
Our Model	43.6	**49.2**	**29.3**
w/o no-object indicator	**43.7**	49.1	29

**Table 5:** Ablation on number of text queries in $Q_{sup}$. Numbers in bold represent the best performance in each metric.

# of Text Queries	PQ	mIoU	AP
# of tokens per sentence (Our Model)	**43.6**	**49.2**	**29.3**
1	42.9	48.4	28.9
Baseline [8]	42.8	49.0	28.7

**Table 6:** Ablation on modality fusion in EQO. Numbers in bold represent the best performance in each metric.

Fusion Method	PQ	mIoU	AP
w/o Fusion(Our Model)	**43.6**	49.2	**29.3**
Cross-modal Skip-connection [41] ^1^	42.9	49	28.8
Cross-modal Skip-connection [41] ^2^	43.3	**49.3**	28.8

^1^ Single-scale visual feature. ^2^ Multi-scale visual feature.

**Figure A1:**
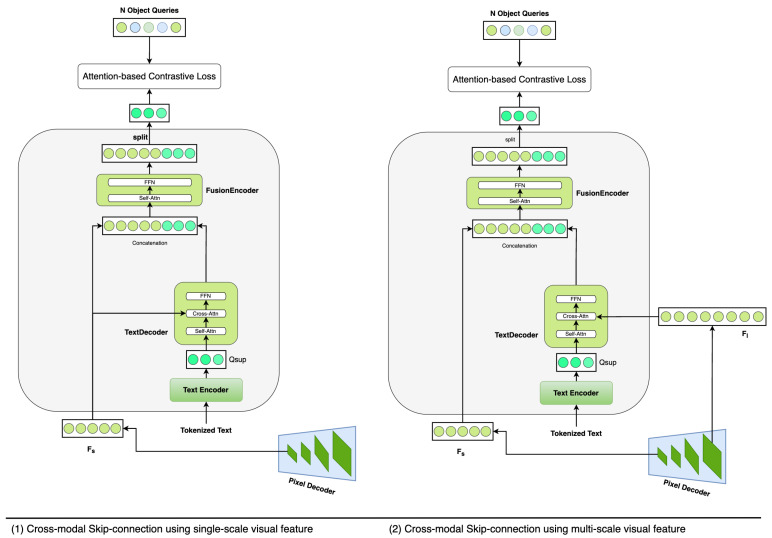
Cross-modal skip-connection network. TextDecoder is composed of a one-layer Transformer Decoder, and FusionEncoder is a one-layer Transformer Encoder. $F_{s}$ denotes the smallest feature map generated by Pixel-decoder, while $F_{l}$ represents the largest one.

**Figure A2:**
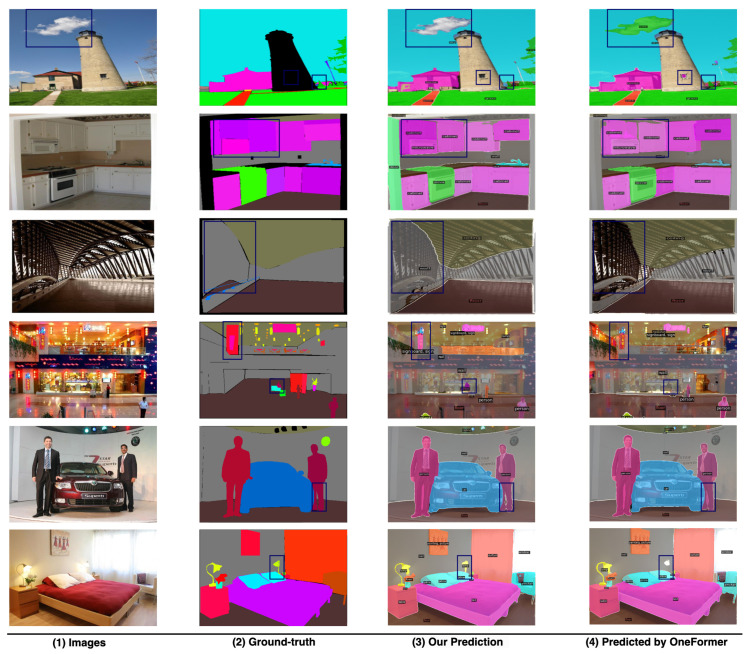
Visualization of panoptic segmentation on ADE20K val.

## Data Availability

No new data were generated for our research; instead, we utilized two public datasets to conduct our experiments (https://www.cityscapes-dataset.com/ (accessed on 31 January 2023) and https://groups.csail.mit.edu/vision/datasets/ADE20K/index.html#Download, accessed on 31 January 2023).

## Appendix A. Cross-Modal Skip-Connection Structure

To find out cross-modality fusion’s impact on $Q_{sup}$ generation in Section 4.4.5, we introduce a cross-modal skip-connection network [41]. This innovative component is designed to bridge the gap between text and visual data, enabling a more holistic understanding of both modalities. There are two prevailing cross-modal fusion network structures; the connected-attention network and the co-attention network. The former applies self-attention to the concatenated visual-text sequence; the latter separately processes two modalities with two transformers [5] and employs the cross-attention modules to realize interactions between two branches. mPLUG [41] combines these two techniques but skips the transformer layers for the visual feature in the co-attention stage.

Different from mPLUG [41], we employ the regular multi-head attention modules instead of the BertAttention [38], which realizes the parameter- and computation-efficiency at the same time. In Figure A1 (1), we illustrate the procedure of two key components in the cross-modal skip-connection network: the Transformer Decoder layer, denoted as the TextDecoder, and the Transformer Encoder layer, referred to as the FusionEncoder. The TextDecoder is responsible for computing the cross-attention between $Q_{sup}$ and the smallest feature map from the pixel-decoder, represented as $F_{s}$. Following this, the FusionEncoder takes over by merging the output of the TextDecoder with $F_{s}$. It then applies the self-attention mechanism to process these combined embeddings. To ensure that the size of the final output matches $Q_{sup}$, we truncate the output of FusionEncoder appropriately.

Further enhancing the performance through modality fusion, we integrate multi-scale visual features into the cross-modality skip-connection network, as shown in Figure A1 (2). Both $F_{s}$ and the largest feature map (denoted as $F_{l}$) are utilized. In this setup, the TextDecoder computes the cross-attention between $Q_{sup}$ and $F_{l}$. Meanwhile, the FusionEncoder concatenates the output from the TextDecoder with $F_{s}$ and executes the self-attention mechanism.

## Appendix B. Visualization on ADE20K val

The sample predictions, as illustrated in Figure A2, are visualized on panoptic segmentation. Our model demonstrates a significant improvement in reducing misclassification, capturing small objects, and generating more precise boundaries when contrasted with the baseline model [8]. The discrepancies in predictions are accentuated using blue rectangular boxes for ease of comparison.
